# Investigating the cell membrane localization of PADI4 in breast cancer cells and inhibition of anti-PADI4 monoclonal antibody

**DOI:** 10.1007/s00432-023-05433-3

**Published:** 2023-10-07

**Authors:** Yan Wang, Xianqin Song, Yu Song, Kehua Fang, Xiaotian Chang

**Affiliations:** 1 Medical Research Center of The Affiliated Hospital of Qingdao University, Wutaishan Road 1677, Qingdao, 266000 Shandong People’s Republic of China; 2 Clinical Laboratory of The Affiliated Hospital of Qingdao University, Wutaishan Road 1677, Qingdao, 266000 Shandong People’s Republic of China

**Keywords:** PADI4, Membrane expression, Molecular therapy, Breast cancer

## Abstract

**Background:**

Peptidyl arginine deiminase 4 (PADI4) is a post-translational modification enzymecan that converts arginine in protein into citrulline in the presence of calcium ions, which is called citrullination. PADI4 has been reported to be expressed in the cytoplasm and nucleus in a variety of malignant tumors. Based on the GeneCards database and our previous research, it is speculated that PADI4 may also be expressed on the cell membrane. This study aimed to confirm the membrane expression of PADI4 and the effect of anti-PADI4 antibodies on cell membrane PADI4. This may be another mechanism of action of anti-PADI4 monoclonal antibodies in the treatment of breast cancer.

**Methods:**

The subcellular localizations of PADI4 in MDA-MB-231 and MCF-7 breast cancer cells were determined by immunofluorescence, immunoelectron microscopy, and Western blot analysis. The tumor cells were treated with PADI4 antibody, and cell proliferation, migration, colony formation, apoptosis, glycolysis, and epithelial-mesenchymal transition (EMT) were measured as well as the expression of some essential tumor genes.

**Results:**

PADI4 was not only localized in the nucleus and cytoplasm of breast cancer cells but was also detected on the cell membrane. Following PADI4 antibody treatment, cell proliferation, migration, colony formation, EMT, and ATP production through glycolysis were decreased, and the mRNA expression of *MYC* proto-oncogene (*MYC*), FAT atypical cadherin 1 (*FAT1*), nuclear factor kappa B subunit 1 (*NFκB*), and tumor necrosis factor (*TNF-α*) in breast cancer cells was downregulated, while the mRNA expression of tumor protein *p63* (*TP63*) was upregulated.

**Conclusions:**

PADI4 is expressed on the cell membrane in breast cancer cells. Anti-PADI4 antibodies can affect the biological functions of cell membrane PADI4, including proliferation, migration, apoptosis, and glycolysis, thereby inhibiting tumor progression.

**Graphical abstract:**

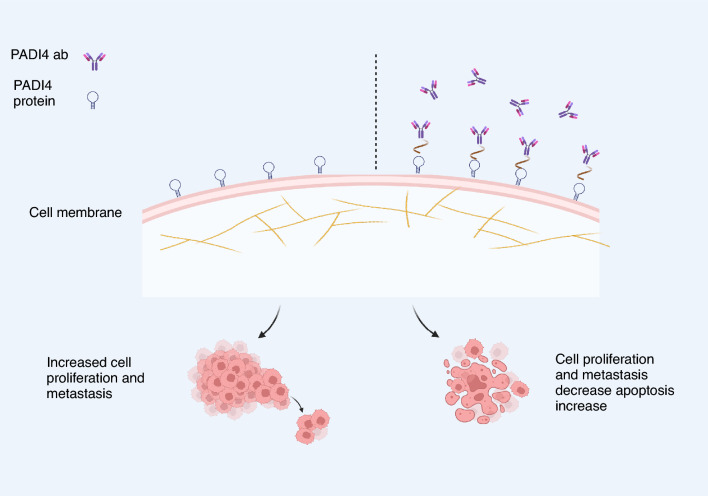

## Introduction

PADI4 (peptidyl arginine deiminase 4) is a member of the PADI family, which was first found in HL-60 cells (Nakashima et al. 1999; Liu et al. 2019a). PADI4 can convert arginine in protein into citrulline in the presence of calcium ions, which is called citrullination. Increased PADI4 expression has been detected in peripheral blood and various tumor tissues, such as gastric cancer, liver cancer, lung cancer, and breast cancer (Chang et al. 2009). PADI4 plays a role in gastric cancer by upregulating the expression of C-X-C motif chemokine receptor 2 (CXCR2), keratin 14 (KRT14), and tumor necrosis factor (TNF-α) (Zheng et al. 2016a). PADI4 also promotes tumorigenesis and metastasis in esophageal squamous cell carcinoma by upregulating carbonic anhydrase 9 (CA9) expression (Liu et al. 2019a). Additionally, PADI4 interacts with Elk-1 at the c-Fos promoter as a transcription factor coactivator in breast cancer (Zhang et al. 2011). PADI4 promotes osteosarcoma cell invasion and migration through epithelial-mesenchymal transition (EMT) (Zhai et al. 2020); many studies have confirmed the carcinogenic role of PADI4 as a cancer susceptibility gene in a variety of cancers. In conclusion, the PADI4 protein exists in a large number of cell and tissue types and plays an important role in key biochemical pathways, which has become a research focus in recent years (Cuthbert et al. 2004; Wang et al. 2004).

Although the role of PADI4 in tumors is well understood, the subcellular localization of PADI4 in tumor cells is unclear. Asaga et al. reported that PADI4 mainly existed in the cytoplasm in eosinophils and neutrophils by immunocytochemical localization (Asaga et al. 2001). Nakashima et al. detected the nuclear localization of PADI4 in HL-60 granulocytes, neutrophils, and eosinophils using immunochemical staining, but no obvious signal was found in the cytoplasm (Nakashima et al. 2002). In cells, the subcellular localization of proteins is closely related to their molecular functions and is associated with a variety of biological and pathological conditions. Proteins in different subcellular compartments may perform different molecular functions within the cell (Liu et al. 2014) Intracellular PADI4 mainly targets histones in the nucleus (Guo et al. 2011; Sorvillo et al. 2019), and citrullination of PADI4 to nuclear histones is thought to be a trigger for the formation of neutrophil extracellular traps (NETs) (Liu et al. 2021). Membrane proteins play an important role in cell survival and intercellular communication, and they are important in signal transduction, energy metabolism, transport processes, and a variety of additional functions that are essential to the survival of organisms. Therefore, they are essential for the formation of drug targets (Rajagopal et al. 2010; Sachse et al. 2014). In previous experiments, we cocultured PADI4 recombinant protein with DC-CIK cells (Liu et al. 2019b) and found that it significantly inhibited tumor growth in tumor-bearing mice by promoting DC maturation, CIK cell proliferation, and cytotoxicity. In previous experiments. PADI4 recombinant protein is a macromolecular substance, which cannot enter cells to play a role but can have a certain effect on cells, suggesting that PADI4 is a target antigen to enhance DC immunotherapy. In addition, the GeneCards database suggests the possibility of PADI4 expression in cell membranes. According to these studies, it was preliminarily speculated that PADI4 might also be expressed on the cell membrane in addition to the cytoplasm and nucleus. Therefore, it is necessary to explore the cell membrane localization of PADI4 and the effect of anti-Padi4 antibodies on the cell membrane of PADI4.

Breast cancer is a common malignant tumor and is the leading cause of cancer death in females (Jafari et al. 2018). PADI4, as a cancer-related gene, is highly expressed in the tissues and blood of breast cancer patients (Chang et al. 2009; Chang and Fang 2010; Stadler et al. 2013). Studies have shown that PADI4 is highly expressed in breast cancer and is a tumor susceptibility gene (Wang et al. 2021; Shi et al. 2020). PADI4 small molecule compound inhibitors (amide and chloramine) can inhibit PADI4 enzyme activity in HL-60, MCF-7, and HT-29 tumor cells, ultimately inhibiting the activation of cancer cells. In addition, PADI4 inhibitor can also effectively inhibit the growth of breast cancer bearing mice (Slack et al. 2011). PADI4 as a target for tumor therapy has been preliminarily studied, proving that PADI4 is a feasible target for tumor therapy (Wang et al. 2012; Song et al. 2020). Small molecule compounds not only have effects on tumor cells but also have certain effects on normal cells. In contrast, monoclonal antibodies have strong specificity and a single biological function. Therefore, we explored the therapeutic effect of anti-PADI4 monoclonal antibodies on breast cancer. In previous experiments, anti-PADI4 monoclonal antibodies were found to inhibit the progression of breast cancer tumors by inhibiting the citrullination of fibronectin in the extracellular matrix (Wang et al. 2022). In this study, we explored the cell membrane localization of PADI4 and whether anti-PADI4 antibody could inhibit the expression of PADI4 in the cell membrane and thus hinder the occurrence and development of tumors, which may be another way for anti-PADI4 antibody to play a therapeutic role in tumor cells outside the cell.

## Materials and methods

### Cell culture

Human breast cancer MDA-MB-231 and MCF-7 cells were obtained from Shanghai Jikai Biotechnology Company (Shanghai, China). The cells were cultured in medium [Dulbecco’s modified Eagle’s medium (DMEM) with 1% antibiotics (penicillin + streptomycin) and 10% fetal bovine serum (FBS)] at 37 °C in a humidified 5% CO_2_ atmosphere.

### Immunofluorescence assay

MDA-MB-231 and MCF-7 cells were inoculated on cover glasses. The culture medium was removed after the cells adhered to the wall, and the cells were fixed with 4% paraformaldehyde (Solarbio, China) for 30 min. Following a wash with phosphate-buffered saline (PBS), the cells were cultured with 0.1% Triton X-100 (Solarbio, China) to increase the permeability to antibodies for 20 min. The cells were blocked with BSA (Solarbio, China) for 30 min and cultured with PADI4 antibody (AtaGenix, China) overnight at 4 ℃. Following another wash with PBS, the cells were incubated with IgG FITC (Absin, China) in the dark for 1 h. The nuclei were stained with DAPI (Solarbio, China). The cover glass was sealed with a sealing agent (Solarbio, China) and observed under laser confocal microscopy (Nikon, Japan).

Cells were treated with PADI4 antibody (AtaGenix, China) for 24 h. Unbound antibodies were washed off with PBS, and the cells were fixed with 4% paraformaldehyde (Solarbio, China) and 0.1% Triton X-100 (Solarbio, China) transparent cells, sealed with BSA blocking solution (Solarbio, China) and incubated with IgG FITC (Absin, China) in darkness for 1 h. The nuclei were stained with a DAPI solution (Solarbio, China). The cells were observed under a laser confocal microscope (Thermo, USA).

### Immunoelectron microscope assay

MDA-MB-231 and MCF-7 cells were collected, and the cell mass was soaked in immunoelectron microscope fixing solution (Servicebio, China). After fixation, the cells were washed with 0.1 M phosphate buffer (Servicebio, China), dehydrated with alcohol, infiltrated, and embedded in resin. The tissue sections were blocked with 1% BSA (Servicebio, China) at room temperature for 30 min, incubated with PADI4 antibody (AtaGenix, China) at 4 °C overnight, and incubated with 10 nm colloidal gold goat anti-mouse secondary antibody for 2 h (Sigma, USA). The tissue sections were dyed in nickel mesh in 2% uranium acetate-saturated alcohol solution for 8 min without light. The cell structure was observed, and images were collected under a transmission electron microscope (Hitachi, Japan).

### Protein extraction and western blot analysis

Cell membrane proteins and cytoplasmic proteins were extracted with a protein extraction kit (Beyotime, China). MDA-MB-231 and MCF-7 cells were collected, washed, and resuspended in PBS. One milliliter of membrane protein extraction reagent A (Beyotime, China) was added to the cells and placed in an ice bath for 15 min. The cell suspension was transferred to a glass homogenizer for approximately 40 homogenizations. After centrifugation at 4 °C and 14,000×*g* for 30 min, the supernatant was collected as cytoplasmic protein. Then, 200 µl of membrane protein extraction reagent B (Beyotime, China) was added. After suspension, the samples were placed in an ice bath for 10 min, followed by centrifugation at 4 °C and 14,000×*g* for 5 min, and the supernatant was collected as the membrane protein solution.

MDA-MB-231 and MCF-7 cells were collected, and RIPA lysis buffer (Elabscience, China) was added after washing with PBS. The cell suspension was collected after 30 min on ice, followed by centrifugation at 12,000 rpm at 4 °C for 10 min, and the supernatant collected was the cell whole protein solution.

Samples (30 µg in each lane) were separated by 12% twelve alkyl sulfate polyacrylamide gel electrophoresis (SDS-PAGE) and transferred to polyvinylidene fluoride (PVDF) membranes. The PVDF membrane was blocked with 5% skim milk, and PADI4 antibody (AtaGenix, China), N-cadherin antibody (CST, USA), Vimentin antibody (CST, USA), and Claudin-1 antibody (CST, USA) were incubated with the membrane at 4 °C overnight. Images of Western blots were captured using an Enhanced Chemiluminescence (ECL) reagent (Affinity, China).

### Cell viability assay

Logarithmically growing MDA-MB-231 and MCF-7 cells were collected. The cell density was adjusted to 3 × 10^3^ cells/well and inoculated on 96-well plates (Corning USA) in an incubator containing 5% CO_2_ at 37 ℃ for 24 h. After adhering to the wall, the cells were treated with 5, 10, 20, or 40 ng/μl PADI4 antibody (AtaGenix, China), and cell proliferation was detected at 0, 12, 24, and 48 h. Ten microliters of Cell Counting Kit-8 (CCK8) reagent (MCE, USA) were added to the cultured cells 2 h before the test. Following incubation in the dark, the absorbance of the cells at 450 nm was measured using a spectrophotometer (Infinite M Plex, Tecan, Switzerland).

### Cell proliferation assay

MDA-MB-231 and MCF-7 cells were collected and counted, and the cell density was adjusted to 1 × 10^5^ cells/ml. The cell suspension was added to the wells of E-Plate 16 (Agilent, USA). After cell adhesion, the cells were treated with PBS or 5, 10, or 20 ng/μl PADI4 antibody (AtaGenix, China). E-Plate 16 was placed into a Real Time CelI AnaIysis (RTCA) DPlus station (Agilent, USA), and cell proliferation was continuously measured for 24 h.

### EdU incorporation assay

MDA-MB-231 and MCF-7 cells were inoculated on a cover glass. After the cells adhered to the wall, the cells were treated with PBS and 10 ng/μl PADI4 antibodies and cultured in the incubator for 24 h. The cells were incubated with 0.1% Triton X-100 (Solarbio, China) for 20 min. Following PBS washes, EdU staining reaction solution (Servicebio, China) was added, and the cells were dyed with DAPI dye solution (Servicebio, China) in the dark for 10 min. After sealing with a sealing agent, the image was observed under an upright fluorescence microscope (Nikon, Japan).

### Colony formation assay

MDA-MB-231 and MCF-7 cells were collected and inoculated on a 6-well plate (Corning USA). After the cells adhered to the wall, they were treated with PBS or 5 or 10 ng/μl PADI4 antibody (AtaGenix, China) for 24 h, and the cells were continually cultured in an incubator for 2 weeks, during which the culture medium was changed regularly. After 2 weeks, the cells were fixed with methanol for 15 min and stained with 0.1% crystal violet (Solarbio China) for 10 min. The cells were photographed and counted.

### Cell migration assay

The cell migration of MDA-MB-231 and MCF-7 cells was measured with 24-well Transwell chambers (Corning USA). The cells were resuspended in a serum-free medium. Then, 500 μl of medium containing 20% FBS was added to the lower Transwell chamber, and 200 μl of serum-free cell suspension was added to the upper Transwell chamber. The cells were treated with PBS and 5 or 10 ng/μl PADI4 antibodies (AtaGenix, China) and cultured in 5% CO_2_ at 37 ℃. After 48 h of culture, the cells that did not pass through the membrane in the upper chamber were wiped with a cotton swab and then fixed with methanol for 30 min, stained with 0.1% crystal violet (Solarbio China) for 15 min, and then photographed and counted under the EVOS M5000 cell imaging system (Thermo, USA).

### RTCA DPlus station migration analysis

An RTCA DPlus station (Agilent, USA) was used to quantitatively analyze the effect of the PADI4 antibody on the migration of MDA-MB-231 and MCF-7 cells. The cells were cultured in 24-well plates (Corning USA). After the cells adhered to the wall, they were treated with PBS or 5 or 10 ng/μl PADI4 antibody. After 24 h of incubation, the cells were collected to prepare a serum-free cell suspension. Then, 100 µl of serum-free cell suspension was added to the upper chamber of a cell invasion and migration (CIM) chamber (Agilent, USA), and 165 µl of medium containing 20% FBS was added to the lower chamber of CIM. CIM was measured with an RTCA DPlus station for 72 h.

### Cell apoptosis assay

Cell apoptosis was detected by an Annexin V-fluorescein isothiocyanate (FITC)/propidium iodide (PI) apoptosis detection kit (Elabscience, USA). The cells were inoculated in 12-well plates and treated with 5 or 10 ng/μl PADI4 antibody for 24 h. The cells were collected and washed twice with PBS and resuspended with 1 × Annexin V Binding Buffer as a solution. Then, 2.5 µl of Annexin V-FITC and 2.5 µl of PI staining solution were added, and the cells were incubated in the dark for 20 min. Then, 400 μl of 1 × Annexin V binding buffer was added, and cell apoptosis was analyzed using flow cytometry (Apogee A50, NovoCyte D2040R, UK).

### Cellular energy metabolism assay

MDA-MB-231 and MCF-7 cells were cultured in XFp cell culture plates (Agilent, USA) in CO_2_ cell culture boxes overnight at 37 °C. The probe plate (Agilent, USA) was added to the XF calibrant (Agilent, USA) in a CO_2_-free cell culture box overnight at 37 °C. PADI4 antibody (5 and 10 ng/μl) was added to XFP cell culture plates, and the cells were treated for 24 h. The culture medium in the XFp cell culture plate was discarded, the detection solution (including DMEM, pyruvate, glucose, and glutamine) was added, and the XFp cell culture plate was placed in a CO_2_-free cell culture box at 37 °C for 1 h. Then, 20 µl of test solution containing Rot/AA (Agilent, USA) was added to the probe plate dosing hole A, and 22 µl of test solution containing oligomycin (Agilent, USA) was added to hole B before getting on the machine. After the probe plate was calibrated in the Agilent Seahorse XFp system (Agilent, USA), the energy metabolism of the treated cells in the XFp cell culture plate was detected. The data were analyzed by the Seahorse Analytics website (https://seahorseanalytics.agilent.com).

### Quantitative real-time PCR assay

RNA was extracted from MDA-MB-231 and MCF cells with RNAiso Plus (TaKaRa, Japan). The extracted RNA was reverse transcribed into cDNA with HiScript III RT SuperMix (Vazyme, China). cDNA was added to ChamQ Universal SYBR qPCR Master Mix (Vazyme, China), and mRNA expression was examined by fluorescence-based real-time quantitative PCR using a LightCycle96 (Switzerland). GAPDH was used as the internal reference control, and the relative mRNA expression was analyzed using the 2^–ΔΔCT^ calculation method. The primer sequences were designed as follows and were synthesized by Sangon Biotech (China).

**Table:** 

Gene	Primer	Sequence
*MYC*	Forward primer	5′- CCTGGTGCTCCATGAGGAGAC -3′
	Reverse primer	5′- CAGACTCTGACCTTTTGCCAGG -3′
*NFκB*	Forward primer	5′- GCAGCACTACTTCTTGACCACC -3′
	Reverse primer	5′- TCTGCTCCTGAGCATTGACGTC -3′
*FAT1*	Forward primer	5′- ATCTGTGGAGCCTCCTGGCATA-3′
	Reverse primer	5′- CATCTGTAGCCTCGACTGTGAG-3′
*TP63*	Forward primer	5′- CAGGAAGACAGAGTGTGCTGGT-3′
	Reverse primer	5′- AATTGGACGGCGGTTCATCCCT-3′
*TNF-α*	Forward primer	5′- CTCTTCTGCCTGCTGCACTTTG-3′
	Reverse primer	5′- ATGGGCTACAGGCTTGTCACTC-3′
*GAPDH*	Forward primer	5′- GTCTCCTCTGACTTCAACAGCG -3′
	Reverse primer	5′- ACCACCCTGTTGCTGTAGCCAA-3′

### Statistical analysis

SPSS 22.0 software and GraphPad Prism 8.0 software were used for statistical analysis. ImageJ 2.1.0 software is used for image analysis. The Student’s *t* test was used to compare the two groups, and the significance of the differences between the groups was assessed using a One-Way ANOVA analysis. An unadjusted *P* value of less than 0.05 was considered significant.

## Results

### Subcellular localization of PADI4 in breast cancer cells

**Fig. 1:**
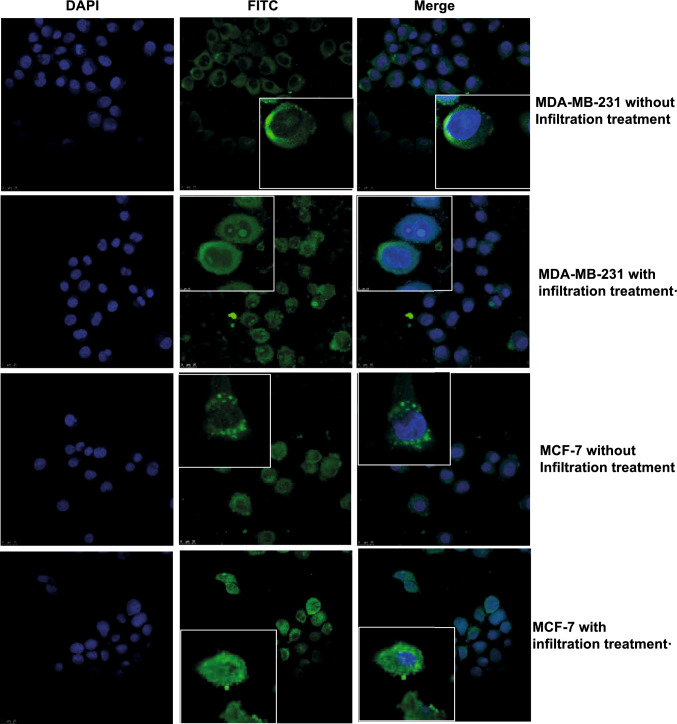
Subcellular localization of PADI4 in MDA-MB-231 and MCF-7 cells was determined by immunofluorescence

**Fig. 2:**
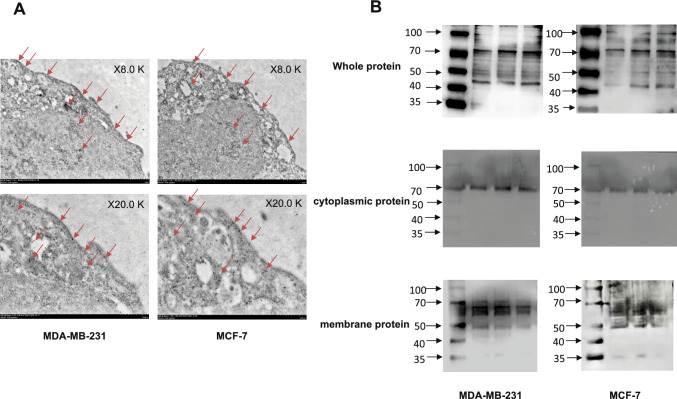
**A** Subcellular localization of PADI4 in MDA-MB-231 and MCF-7 cells determined by immunoelectron microscopy. **B** Subcellular expression of PADI4 in MDA-MB-231 and MCF-7 cells. Total protein, cytoplasmic protein, and membrane protein were analyzed using Western blot analysis with PADI4 antibody

The subcellular localization of the PADI4 protein in MDA-MB-231 and MCF-7 cells was examined by immunofluorescence. In the absence of a permeable membrane, most PADI4 antibody immune signals were detected on the cell membrane surface. After the membrane penetration, the immunosignal of the PADI4 antibody was observed in the nucleus, cytoplasm, and membrane (Fig. 1). We also observed the subcellular localization of PADI4 in MDA-MB-231 and MCF-7 cells using immunoelectron microscopy. The immune signals of colloidal gold particles were detected in the cell membrane, cytoplasm, nucleus, and other organelles (Fig. 2A). Total proteins were extracted from the whole cell, cytoplasm, or membrane of cultured MDA-MB-231 and MCF-7 cells. Western blotting was performed to detect PADI4 expression using the PADI4 antibody. An immunosignal with a molecular weight of approximately 75 kDa and an immunosignal with a molecular weight of approximately 45 kDa were detected in the whole protein; an immunosignal with a molecular weight of approximately 72 kDa was detected in the total protein from cytoplasmic sections; and an immunosignal with a molecular weight of approximately 67 kDa and an immunosignal with a molecular weight of approximately 48 kDa were detected in the total protein from cell membranes (Fig. 2B).

### Effect of anti-PADI4 antibody on cell viability

**Fig. 3:**
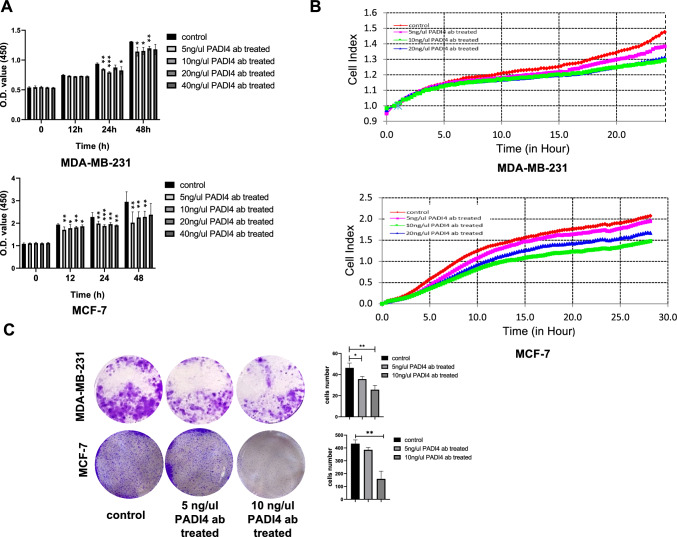
The effect of the PADI4 antibody on the cell viability of MDA-MB-231 and MCF-7 breast cancer cells. **A** The effect of the PADI4 antibody on cell proliferation detected by CCK-8 assays. **B** The effect of the PADI4 antibody on cell proliferation detected by RTCA assays. **C** The effect of the PADI4 antibody on cell clonal formation. ^*^P < 0.05, ^**^P < 0.01, ^***^P < 0.001

**Fig. 4:**
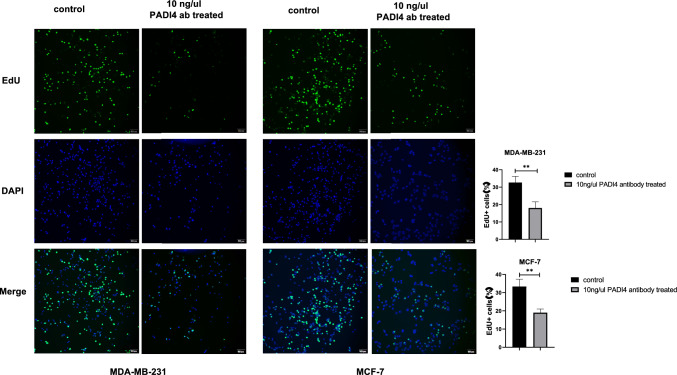
The effect of the PADI4 antibody on the viability of MDA-MB-231 and MCF-7 breast cancer cells determined using an EdU assay. ^*^P < 0.05, ^**^P < 0.01, ^***^P < 0.001

To detect the effect of the PADI4 antibody on the viability of breast cancer cells, we performed a CCK-8 assay to assess cell growth. After MDA-MB-231 and MCF-7 cells were treated with PADI4 antibody at different concentrations for 12, 24, and 48 h, the cell viability decreased, indicating inhibition of cell proliferation by the PADI4 antibody. The cell viability decreased most significantly when the cells were treated with 10 ng/μl antibody for 24 h (*P* < 0.001, *P* < 0.001) (Fig. 3A). The RTCA DPlus station was also adopted to detect cell proliferation. After treatment with the PADI4 antibody, the viability of MDA-MB-231 and MCF-7 cells was inhibited, and their proliferation decreased compared with that of the control group without treatment (Fig. 3B). Additionally, cell viability was proven by cell colony formation analysis. Compared with that of the normal control group, the colony formation ability decreased following PADI4 antibody treatment, which was significant in the presence of 10 ng/μl antibody (*P* < 0.01, *P* < 0.01) (Fig. 3C). Furthermore, the effect of the PADI4 antibody on the viability of breast cancer cells was proven using an EdU incorporation experiment. Compared with that of the normal controls without treatment, the proportion of EdU + cells and DNA replication of MDA-MB-231 and MCF-7 cells decreased following treatment with the PADI4 antibody (*P* < 0.01, *P* < 0.01) (Fig. 4).

### Effect of anti-PADI4 antibody on cell migration

**Fig. 5:**
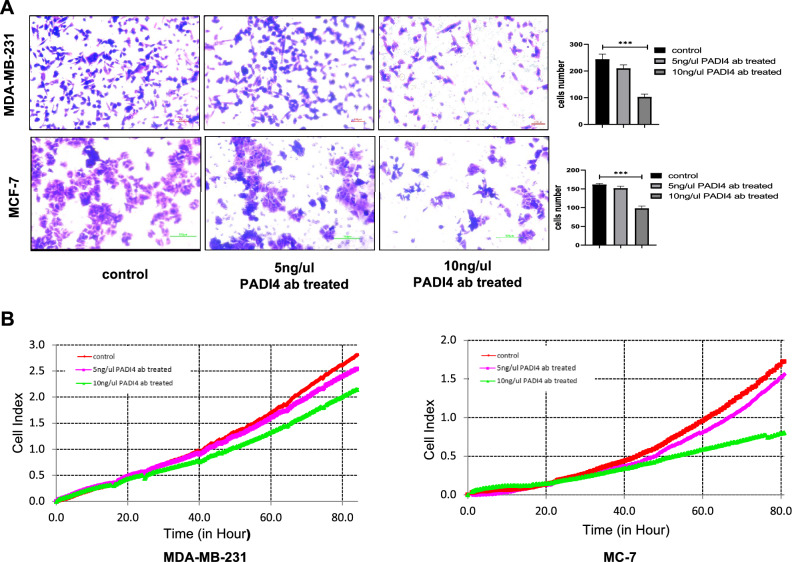
The effect of the PADI4 antibody on breast cancer cell migration of MDA-MB-231 and MCF-7 cells. **A** Cell migration detected by Transwell assays. **B** Cell migration was detected using the RTCA DPlus station. ^*^P < 0.05, ^**^P < 0.01, ^***^P < 0.001

The effect of the PADI4 antibody on the migration of breast cancer cells was examined by Transwell assays. After 24 h of treatment with different concentrations of PADI4 antibody, the migration of MDA-MB-231 and MCF-7 cells decreased, and antibody treatment at a concentration of 10 ng/μl showed the most significant effect (*P* < 0.001, *P* < 0.001) (Fig. 5A). The RTCA DPlus station was also adopted to measure the inhibitory effect of the PADI4 antibody on the migration of breast cancer cells. Compared with that of the control cells, the migration of MDA-MB-231 and MCF-7 cells decreased following PADI4 antibody treatment (Fig. 5B**)**.

### Effect of anti-PADI4 antibody on cell apoptosis and energy metabolism

**Fig. 6:**
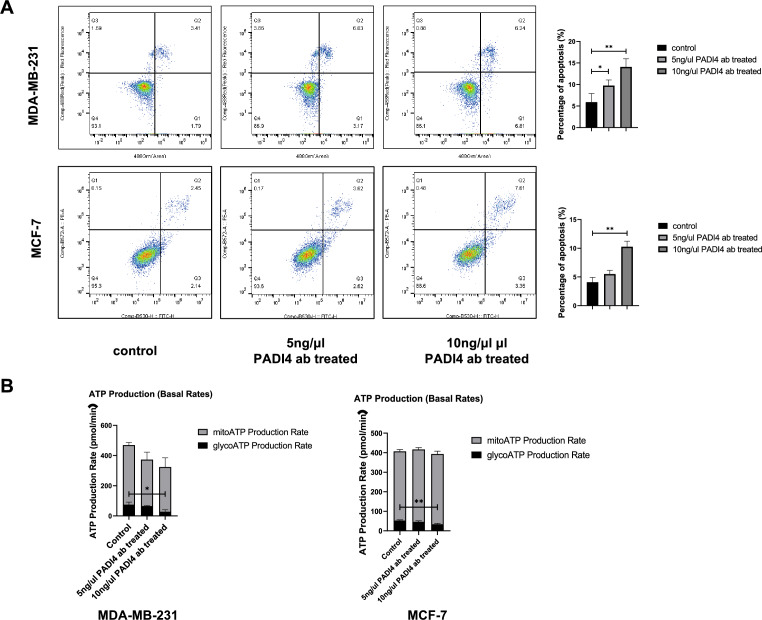
The effect of PADI4 antibody on breast cancer cell apoptosis and ATP production in MDA-MB-231 and MCF-7 cells. A. Apoptosis detected using flow cytometry. B. ATP production by mitochondrial respiration and glycolytic respiration in the cells detected using an Agilent Seahorse XFp system. ^*^P < 0.05, ^**^P < 0.01, ^***^P < 0.001

To explore the effect of the PADI4 antibody on the apoptosis of MDA-MB-231 and MCF-7 breast cancer cells, we conducted flow cytometry. Compared with that of the control group, apoptosis increased after treatment with different concentrations of PADI4 antibody. The apoptosis of MDA-MB-231 cells treated with the antibody at a concentration of 5 ng/μl increased (*P* < 0.05), and the apoptosis of MDA-MB-231 and MCF-7 cells treated with the antibody at a concentration of 10 ng/μl was the most significant (*P* < 0.01, *P* < 0.01) (Fig. 6A).

Cell energy metabolism was detected by an Agilent hippocampal energy XFP system. There was no significant difference in ATP produced by mitochondria between the normal control cells and the cells treated with the PADI4 antibody. Compared with that of the normal control cells, ATP produced by glycolysis decreased after PADI4 antibody treatment, especially in the cells treated with 10 ng/μl PADI4 antibody (*P* < 0.05, *P* < 0.01) (Fig. 6B).

### Effect of anti-PADI4 on cell EMT

**Fig. 7:**
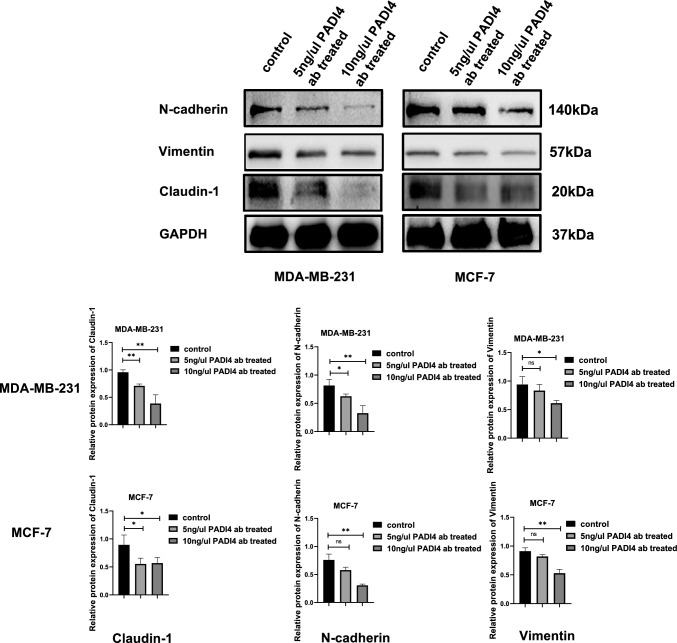
The effects of the PADI4 antibody on EMT markers in breast cancer cells.^*^P < 0.05, ^**^P < 0.01, ^***^P < 0.001

The expression of key EMT proteins in breast cancer cells was examined using Western blotting. Compared with that of the normal control cells without treatment, the protein expression of N-cadherin (*P* < 0.05, *P* = 0.0542), Vimentin (*P* = 0.3587, *P* = 0.083), and Claudin-1 (*P* < 0.01, *P* < 0.05) in MDA-MB-231 and MCF-7 cells decreased after treatment with 5 ng/µl PADI4 antibody. After treatment with 10 ng/µl PADI4 antibody, the protein expression of N-cadherin (*P* < 0.01, *P* < 0.01), Vimentin (*P* < 0.05, *P* < 0.01), and Claudin-1 (*P* < 0.01, *P* < 0.05) in MDA-MB-231 and MCF-7 cells further decreased (Fig. 7).

### Effect of anti-PADI4 on important target genes in cells

**Fig. 8:**
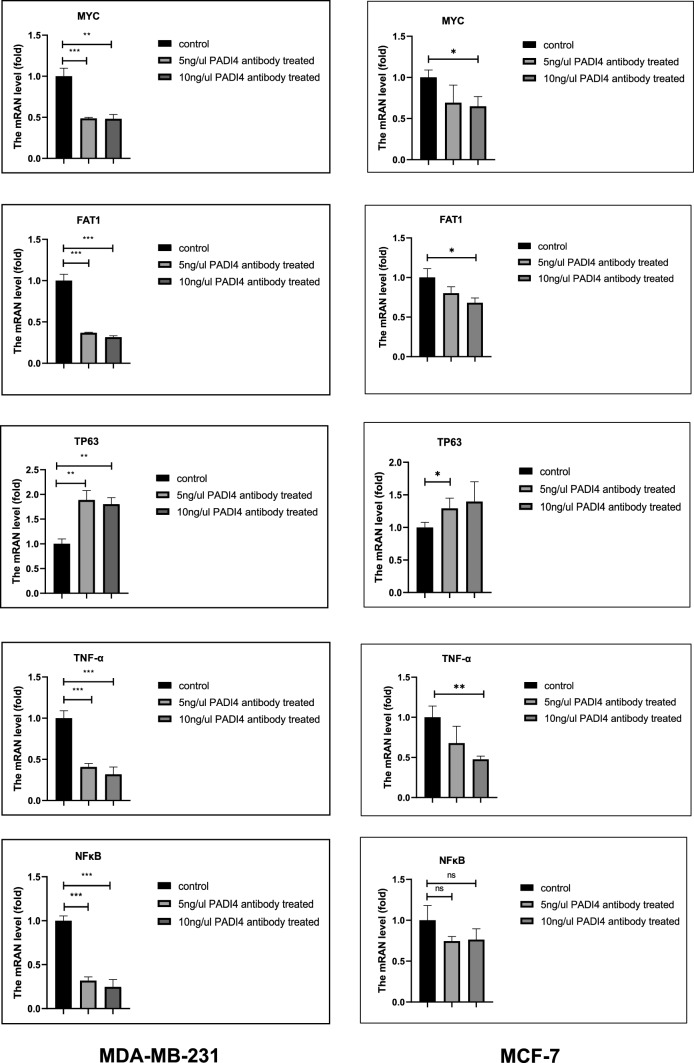
The effects of the PADI4 antibody on *MYC*, *FAT1*, *NFκB*, *TNF-α* and *TP63.* mRNA expression using real-time PCR. ^*^P < 0.05, ^**^P < 0.01, ^***^P < 0.001

The mRNA expression of tumor-related key genes in the MDA-MB-231 and MCF-7 cells treated with PADI4 antibody was examined using qRT-PCR. Compared with those of the normal cells without PADI4 antibody treatment, the mRNA levels of *MYC* (*P* < 0.001, *P* < 0.01), *FAT1* (*P* < 0.001, *P* < 0.001), *TNF-α* (*P* < 0.001, *P* < 0.001) and *NFκB* (*P* < 0.001, *P* < 0.001) decreased and the mRNA level of *TP63* (*P* < 0.01, *P* < 0.01) increased in the MDA-MB-231 cells treated with 5 ng/μl and 10 ng/μl PADI4 antibodies. After treatment with 5 ng/μl PADI4 antibody, the mRNA level of *TP63* in MCF-7 cells increased (*P* < 0.05), and the mRNA levels of *MYC* (*P* < 0.05), *FAT1* (*P* < 0.05) and *TNF-α* (*P* < 0.01) in MCF-7 cells decreased after treatment with 10 ng/μl PADI4 antibody (Fig. 8).

## Discussion

In this study, the subcellular localization of PADI4 in breast cancer cells was examined by immunofluorescence and immunoelectron microscopy. The results showed that PADI4 was expressed not only in the cytoplasm and nucleus but also in the cell membrane. Western blot analysis also detected differential molecular weights of PADI4 in total proteins from whole cells, cell cytoplasm, and cell membranes in breast cancer cells. An immunosignal of PADI4 at a molecular weight of approximately 67 kDa was detected in protein samples that were extracted from the cell membranes. The above observations identified PADI4 protein expression in the tumor cell membrane.

To date, over 400 papers about PADI4 have been published, but the subcellular localization of PADI family members is not completely clear. PADI1, PADI2, PADI3, PADI4, and PADI6 were reported to be expressed in the nucleus and cytoplasm (Cuthbert et al. 2004; Guerrin et al. 2003; Ishigami et al. 2002; Basmanav at al. 2016; Xu et al. 2016). PADI2 and PADI4 were also detected in extracellular and euchromatin regions (Ishigami et al. 2002; Foulquier et al. 2007; Zhang et al. 2012). Arita et al. proposed that PADI4 is expressed in some protein-containing complexes (Arita et al. 2004). Various types of PADI have been reported to be closely related to the insoluble membrane in normal mouse brain tissue (Hidenari et al. 1987; Moscarello et al. 2002; Musse et al. 2008), and PADI2 located on various insoluble membranes can rapidly and efficiently citrullinize intermediate filaments and membrane-binding proteins. Jang et al. detected the subcellular localization of PADI2 in the brains of scrapie-infected mice using immunoelectron microscopy and proposed that immunogold-labeled PADI2 is expressed in many membrane-containing organelles, such as mitochondria, microsomes, and glial filaments, which may be related to Ca^2^ + flow between various membrane-containing organelles and the plasma membrane (Jang et al. 2011). In addition, the GeneCards database indicates that PADI4 might exist in many organelles, such as the cytoplasm, nucleus, extracellular space, cell membrane and mitochondria, and the confidence is 5, 5, 3, 2, and 2 and the Z score is 3.7, 4.6, 5.2, 4.2 and 3.5, respectively (https://www.genecards.org/cgibin/carddisp.pl?gene=PADI4&keywords=PADI4). Our previous experiments cultured DC-CIK cells with recombinant PADI4 protein and found that recombinant PADI4 protein promoted DC maturation and CIK cell proliferation, suggesting that PADI4 is a target antigen for enhanced DC immunotherapy (Liu et al. 2019b). PADI4 recombinant protein is a macromolecular substance that is difficult to enter cells to play a role but has an effect on immune cells and tumor cells. Combined with the results of this study, it is further confirmed that PADI4 may be expressed on the cell membrane of breast cancer cells.

The different molecular weights of the PADI4 protein have been reported in many articles. Our previous studies showed that PADI4 has a molecular weight of 67 kDa in esophageal cancer (Chang et al. 2011), 74 kDa in esophageal squamous cell carcinoma (Liu et al. 2019a), and 74 kDa (Chang et al. 2022) and 70 kDa in gastric cancer (Zheng et al. 2016a). Kolodziejs et al. detected PADI4 with a molecular weight of 70 kDa in leukemia cells (Kolodziej et al. 2014), Guo et al. detected PADI4 with a molecular weight of 74 kDa in osteosarcoma (Guo et al. 2021), Hollingsworth et al. detected PADI4 with a molecular weight of 65 kDa in mouse retinal cells (Hollingsworth et al. 2018), and Uysal-Onganer et al. detected PADI4 with a molecular weight of 25 kDa in human pancreatic cancer cells (Uysal-Onganer et al. 2021). PADI4 shows different molecular weights in different studies, and even the molecular weight of PADI4 is not completely the same in the same tumor cells (Liu et al. 2019a; Zheng et al. 2016a; Chang et al. 2011; Chang et al. 2022; Hollingsworth et al. 2018; Uysal-Onganer et al. 2021). Western blot analysis in some studies also showed multiple PADI4 protein bands, which may be related to the differential subcellular localizations of PADI4 in these cells. Based on our study with proteins that were extracted from different cell locations, we suggest that PADI4 has a molecular weight of approximately 72 kDa in the cytoplasm and 65–67 kDa in the cell membrane.

Subcellular localization can locate proteins to specific subcellular structures in cells, providing research directions for understanding the mechanism of protein action. The subcellular localization of proteins has attracted many people’s attention, different locations of proteins may lead to different biological functions, it can be determined through molecular biotechnology, and this subcellular localization is of great significance (Martinez-Val et al. 2021; Sirover 2012; Alvarez-Paggi et al. 2017). We investigated the inhibitory effect of anti-PADI4 antibody on the biological function of cell membrane PADI4. MDA-MB-231 and MCF-7 cells are treated with anti-PADI4 antibody. Antibodies usually cannot enter the cells and can only act by binding to targets on the cell membrane. Therefore, anti-PADI4 antibody can act by targeting and binding to the PADI4 protein on the cell membrane. We investigated the effects of antibodies on cell proliferation, migration, apoptosis, energy metabolism, and EMT.

Compared with normal cells without anti-PADI4 antibody treatment, cell proliferation, migration, and colony formation ability were significantly reduced and apoptosis increased after anti-PADI4 antibody treatment, which was consistent with the results of previous studies on the role of PADI4 in tumor (Liu et al. 2019a; Zheng et al. 2016b). After the anti-PADI4 antibody inhibited the expression of cell membrane PADI4, breast cancer cell growth and metastasis were reduced. Glucose metabolism is the key metabolic pathway of tumor cells, among which aerobic glycolysis is one of the main metabolic modes of tumor (Lebelo et al. 2019). This phenomenon promotes the proliferation and metabolism of tumors and is of great significance in the occurrence and development of tumors. We examined the effect of the anti-PADI4 antibody on the glycolysis of MDA-MB-231 and MCF-7 cells. After treatment with an anti-PADI4 antibody, the proportion of ATP produced by glycolysis in breast cancer cells decreased, and the aerobic glycolysis in the tumor was reduced. It was proved that anti-PADI4 antibody could inhibit the expression of PADI4 on the cell membrane of breast cancer, thus reducing the rate of energy supply by glycolysis in cells, and reducing the metabolism of tumors to a certain extent. EMT is a biological process in which epithelial cells are transformed into mesenchymal cells and plays an important role in chronic inflammation, cancer metastasis, and fibrosis (Bhattacharya and Scime 2019). In this study, breast cancer cells treated with anti-PADI4 antibodies showed decreased expression of EMT-related proteins, including vimentin, claudin-1, and N-cadherin. It has been reported that PADI4 can promote the invasion and migration of osteosarcoma cells through EMT (Zhai et al. 2020), and anti-PADI4 inhibits the EMT of breast cancer cells by inhibiting the expression of PADI4 in the cell membrane.

There is no consensus on the mechanism by which PADI4 plays a role in tumors. Some scholars believe that PADI4 promotes the occurrence and development of gastric cancer by up-regulating *TNF-α*, *CXCR2*, and *KRT14* in *MNK-45* cells and SGC 7901 cells (Zheng et al. 2016b). Abnormal PADI4 expression increases *N-MYC* oncogene expression and promotes cell proliferation in GH3 cells of rat pituitary epithelium (DeVore et al. 2018). In ovarian cancer SKOV3 and A2780 cells, PADI4 regulates cell proliferation, apoptosis, migration, and invasion through the p53 pathway (Cui et al. 2016). *MYC*, *FAT1*, *NF-κB*, *TNF-α*, and *TP63* have been shown to be cancer-related genes and are all associated with the tumor-promoting effects of PADI4 (Zheng et al. 2016b; Park et al. 2015; Duffy et al. 2021; DiDonato et al. 2012; Srivastava et al. 2018) Therefore, we hypothesized that anti-PADI4 antibody could inhibit the expression of *MYC*, *FAT1*, *NFκB*, *TNF-α*, and *TP63* genes by inhibiting cell membrane PADI4, which could inhibit tumor growth, metastasis, and glycolysis.

In summary, this study confirmed that PADI4 is expressed on cell membranes, and membrane-expressed PADI4 stimulates the growth, metastasis and glycolysis of tumor cells, and anti-PADI4 monoclonal antibody can inhibit the biological activity of breast cancer cells by inhibiting the expression of membrane PADI4.

## Data Availability

All data generated or analyzed during this study are included in this published article and its supplementary information files.
